# *Clonorchis sinensis* infection induces pathological changes in feline bile duct epithelium and alters biliary microbiota composition[Fn FN1]

**DOI:** 10.1051/parasite/2024053

**Published:** 2024-09-06

**Authors:** Feng Li, Yanli Zhang, Chunfu Li, Fenqi Li, Baojiang Gan, Hong Yu, Jian Li, Xinyu Feng, Wei Hu

**Affiliations:** 1 College of Life Sciences, Inner Mongolia University Hohhot 010070 PR China; 2 Department of Pathology, Inner Mongolia People’s Hospital Hohhot 010011 PR China; 3 Basic Medicine College, Guangxi Traditional Chinese Medical University Nanning 530005 Guangxi PR China; 4 One Health Center, Shanghai Jiao Tong University-The University of Edinburgh Shanghai 20025 PR China; 5 School of Global Health, Chinese Center for Tropical Diseases Research, Shanghai Jiao Tong University School of Medicine Shanghai 20025 PR China; 6 Department of Infectious Diseases, Huashan Hospital, State Key Laboratory of Genetic Engineering, Ministry of Education Key Laboratory for Biodiversity Science and Ecological Engineering, Ministry of Education Key Laboratory of Contemporary Anthropology, School of Life Sciences, Fudan University Shanghai 200438 PR China

**Keywords:** *Clonorchis sinensis*, Biliary microbiota, Full-length 16S rRNA gene sequencing, *Streptococcus*

## Abstract

*Background: Clonorchis sinensis* is a zoonotic liver fluke that inhabits the bile ducts of the human liver for prolonged periods, leading to cholangiocarcinoma. Recent research indicates associations between altered biliary microbiota and bile duct disorders. However, the impacts of *C. sinensis* infection on bile duct epithelium and subsequent effects on biliary microbiota remain unknown. *Methods:* Feline bile duct samples were collected from both uninfected and *C. sinensis*-infected cats. Histopathological examination was performed to assess epithelial changes, fibrosis, mucin and cell proliferation using hematoxylin-eosin staining and immunohistochemistry. Additionally, biliary microbiota composition was analyzed through 16S rRNA gene sequencing. Statistical analyses were conducted to compare the microbial diversity and relative abundance between infected and uninfected samples. *Results:* Histopathological analysis of infected feline bile ducts revealed prominent epithelial hyperplasia characterized by increased cell proliferation. Moreover, periductal fibrosis and collagen fibrosis were observed in infected samples compared to uninfected controls. Biliary microbial richness decreased with disease progression compared to uninfected controls. *Streptococcus* abundance positively correlated with disease severity, dominating communities in cancer samples. Predictive functional analysis suggested that *C. sinensis* may promote bile duct lesions by increasing microbial genes for carbohydrate metabolism, replication, and repair. *Conclusions:* This study provides comprehensive insights into the pathological effects of *C. sinensis* infection on feline bile duct epithelium and its influence on biliary microbiota composition. These novel findings provide insight into *C. sinensis* pathogenesis and could inform therapeutic development against human clonorchiasis. Further research is warranted to elucidate the underlying mechanisms driving these changes and their implications for host-parasite interactions.

## Abstract (fr)

*Contexte : Clonorchis sinensis* est une douve zoonotique du foie qui habite les voies biliaires du foie humain pendant des périodes prolongées, conduisant au cholangiocarcinome. Des recherches récentes indiquent des associations entre une altération du microbiote biliaire et des pathologies des voies biliaires. Cependant, les impacts de l’infection par *C. sinensis* sur l’épithélium des voies biliaires et les effets ultérieurs sur le microbiote biliaire restent inconnus. *Méthodes :* Des échantillons de voies biliaires félines ont été prélevés sur des chats non infectés et infectés par *C. sinensis*. Un examen histopathologique a été réalisé pour évaluer les modifications épithéliales, la fibrose, la mucine et la prolifération cellulaire à l’aide de la coloration à l’hématoxyline-éosine et de l’immunohistochimie. De plus, la composition du microbiote biliaire a été analysée par séquençage du gène de l’ARNr 16S. Des analyses statistiques ont été menées pour comparer la diversité microbienne et l’abondance relative entre les échantillons infectés et non infectés. *Résultats :* L’analyse histopathologique des voies biliaires félines infectées a révélé une hyperplasie épithéliale importante caractérisée par une prolifération cellulaire accrue. De plus, une fibrose péricanalaire et une fibrose du collagène ont été observées dans les échantillons infectés par rapport aux témoins non infectés. La richesse microbienne biliaire diminue avec la progression de la maladie par rapport aux témoins non infectés. L’abondance des streptocoques est positivement corrélée à la gravité de la maladie, dominant les communautés dans les échantillons avec cancer. L’analyse fonctionnelle prédictive suggère que *C. sinensis* pourrait favoriser les lésions des voies biliaires en augmentant les gènes microbiens pour le métabolisme des glucides, la réplication et la réparation. *Conclusions :* Cette étude fournit des informations complètes sur les effets pathologiques de l’infection à *C. sinensis* sur l’épithélium des voies biliaires félines et son influence sur la composition du microbiote biliaire. Ces nouvelles découvertes donnent un aperçu sur la pathogenèse de *C. sinensis* et pourraient éclairer le développement thérapeutique contre la clonorchiase humaine. Des recherches supplémentaires sont nécessaires pour élucider les mécanismes sous-jacents à l’origine de ces changements et leurs implications sur les interactions hôte-parasite.

## Introduction

Clonorchiasis, an infectious disease caused by the liver fluke *Clonorchis sinensis*, can lead to hepatobiliary conditions, including cholelithiasis, cholangitis, cholecystitis, and cholangiocarcinoma [39]. Previous studies have established correlations between *C. sinensis* infection prevalence and biliary disease incidence, including cholangiocarcinoma [32]. In 2009, the International Agency for Research on Cancer classified *C. sinensis* as a Group 1 carcinogen [55]. Currently, around 5000 cholangiocarcinoma cases annually are attributed to *C. sinensis* infection [40]. However, the precise carcinogenic mechanisms induced by *C. sinensis* remain unclear. In recent years, alterations in the human microbiome have been associated with cancer onset and progression [11, 12, 44, 45, 50], including cholangiocarcinoma [12]. Yet, the role of biliary microbiota changes in *C. sinensis*-induced carcinogenesis is still unknown. Investigations on the biliary microbiota following *C. sinensis* infection may provide insights into carcinogenic pathways triggered by the parasite.

Humans are infected by consuming undercooked freshwater fish or shrimp containing *C. sinensis* metacercariae [26, 29, 56]. The metacercariae excyst in the duodenum and then migrate to the intrahepatic bile ducts. In humans, this leads to bile duct pathology such as the ductular reaction [3] and biliary intraepithelial neoplasia [14]. Prolonged ductular reaction can lead to the development of biliary intraepithelial neoplasia, which can further progress to cholangiocarcinoma. In addition to the histopathological changes, recent research has indicated associations between biliary microbiota changes and cholangiopathies [41]. Compared to healthy individuals, cholelithiasis patients have lower bile α-diversity and a dominance of Actinobacteria, Firmicutes, and Bacteroidetes [16–18]. β-diversity analysis indicates considerable changes in the microbial structure of bile ducts at various clinical phases [10, 24, 28]. In cholangiocarcinoma patients, *Enterobacter*, *Pseudomonas*, and *Stenotrophomonas* dominate versus gallstone patients with higher Shannon diversity [5, 42]. In primary sclerosing cholangitis, Firmicutes, Gemellales, Actinobacteria, Bacteroidetes, and Fusobacteria increase with inflammation duration [42]. Together, these findings suggest a potential role of the biliary microbiota in cholangiopathies.

As the definitive host for *C. sinensis*, cats play a crucial role in the zoonotic transmission of this pathogen to humans. Previous research has established that among various mammals, only humans and cats are known to naturally develop bile duct cancer as a result of *C. sinensis* infection. By contrast, experimental models in other mammals, such as Syrian golden hamsters and rats, necessitate the introduction of chemical carcinogens like N-nitrosodimethylamine [54] and diethylnitrosamine [38], respectively, to induce cholangiocarcinoma. Additionally, *C. sinensis* infections in cats and their correlation with bile duct pathology are poorly understood. The epithelial and adenomatous hyperplasia observed in cats closely reflects the ductular reactions and biliary intraepithelial neoplasia seen in humans. This study aimed to elucidate the distinctions in hepatobiliary histopathology and biliary microbiota between healthy and *C. sinensis*-infected feline subjects. Furthermore, we sought to determine potential correlations between these microbiotic changes and progression of the disease. Insights derived from this study could provide a foundational understanding of clonorchiasis, potentially guiding the development of novel strategies for the treatment and prevention of this disease in animals and humans.

## Materials and methods

### Ethics

The felines used in this study were sourced from the Nanning Animal Shelter. They were humanely euthanized by certified veterinarians via lethal injection, adhering to standardized protocols. This action was taken primarily due to the animals’ limited chances of adoption, not specifically for research purposes. Post-euthanasia, all samples were promptly collected. The process was ethically vetted and approved by the Ethics Committee of Inner Mongolia University, as documented in the Inner Mongolia University Bioethics Document No. [2023]051.

### Sample collection and grouping

Over a three-year period (2021–2023), a total of 112 feline liver samples were examined at the Nanning Animal Shelter in Guangxi, China. Through an inclusion screening process, 26 cases were selected for analysis based on the absence of other concurrent infections, as determined by systematic examination of visceral organs, which showed no significant abnormalities. The remaining 86 samples had varying degrees of visceral illness, including substantial pulmonary edema (*n* = 22), pneumonia (*n* = 27), and erosion or ulcers of the intestines or stomach (*n* = 37). The included samples were then categorized into two groups based on the presence or absence of *C. sinensis* infection, as confirmed through autopsy findings and the absence of eggs or adult parasites in bile or bile ducts. The uninfected group (*n* = 8) showed no signs of infection or pathological changes in other visceral organs. The infected group (*n* = 18) was identified by the presence of *C. sinensis* in the bile ducts or gallbladder, with further subdivisions into epithelial hyperplasia (*n* = 7, EH), adenomatous hyperplasia (*n* = 8, AH), and carcinoma (*n* = 3, CA) groups based on histological analysis using hematoxylin and eosin (HE) staining of liver sections. Bile samples were collected post-mortem from the gallbladder by puncture and stored at −80 °C until further analysis [54].

### Immunohistochemistry and histopathology

Liver sections were subjected to sanitation with 75% ethanol and subsequently dissected to procure bile duct tissues from both uninfected cats and those exhibiting *C. sinensis* infection. The obtained tissues were then fixed overnight in 4% paraformaldehyde, dehydrated through an ethanol series, and embedded in paraffin. Subsequently, sections of 6 μm thickness were prepared using a Leica cryostat. These sections were then subjected to staining with hematoxylin and eosin (HE), Masson’s trichrome, Sirius red, Alcian blue, cytokeratin-7 (CK7), and proliferative cell nuclear antigen (PCNA) for histopathological examination.

### DNA extraction, PCR amplification, and sequencing

Total DNA was extracted from samples using an MP Biomedicals FastDNA^TM^ SPIN Kit for Soil, per the manufacturer’s instructions. DNA integrity was verified by 1% agarose gel electrophoresis and purity by spectrophotometry (NanoDrop 2000, Thermo Fisher Scientific, Waltham, MA, USA). The 16S rRNA gene was amplified by PCR using barcoded primers 27F and 1492R (10 ng template DNA, 27 cycles). Amplicons were verified by 2% agarose gel electrophoresis and purified with paramagnetic beads (AMPure PB; Pacific Biosciences, Menlo Park, CA, USA). Purified amplicons were quantified by fluorometry (Quantus, Promega, Madison, WI, USA) and pooled equimolarly. A DNA library was constructed using a SMRTbell prep kit 3.0 (Pacific Biosciences) and sequenced on a PacBio Sequel IIe system (Majorbio, Shanghai, China).

### Sequence processing

Raw PacBio subreads were converted to high-fidelity (HiFi) circular consensus sequences (CCS) using SMRTLink v11.0 software (Pacific Biosciences) with parameters minFullPass = 3 and minPredictedAccuracy = 0.99. CCS were demultiplexed by sample barcode and primer sequences trimmed using the cutadapt v2.5 utility. Amplicon sequence variants (ASVs) were inferred using the DADA2 algorithm (v1.16) in QIIME2 (v2019.10) with default parameters. DADA2 filters sequences, corrects errors, dereplicates, denoises, merges paired ends, and constructs the ASV table. Taxonomy was assigned to ASVs using a naïve Bayes classifier trained on the Silva v138 99% OTUs reference taxa in the 515F/806R region of the 16S rRNA gene. Chloroplast and mitochondrial sequences were identified by taxonomy and removed. Samples were rarefied to an equal depth of 19,384 sequences per sample to minimize the impact of sequencing depth on downstream analyses. Good’s coverage was calculated to confirm thorough sequence coverage (average 99.09% ± 0.05% across samples). The datasets are available on the Biotechnological Information (NCBI) Short Read Archive (SRA) under accession number PRJNA1033204. The accession numbers for the individual run files are SAMN38028082–SAMN38028107.

### Microbiota analysis

In order to understand the impact of *Clonorchis sinensis* infection on the host’s biliary microbiota, alpha diversity metrics, including observed ASVs, Chao1 richness estimator, and Shannon diversity index were calculated in Mothur v1.43.0 and compared between two groups using the nonparametric Wilcoxon rank-sum and four groups using the nonparametric Kruskal–Wallis tests. To further characterize differences in microbiota composition between groups, β-diversity was assessed using the abundance-based Jaccard dissimilarity metric and visualized by non-metric multidimensional scaling (NMDS) ordination in QIIME2. Differences in community structure were tested using analysis of similarities (ANOSIM). Differential abundance analysis was performed to identify ASVs and genera enriched between uninfected and infected groups using the Wilcoxon rank-sum test and Benjamin-Hochberg false discovery rate correction. Linear discriminant analysis effect size (LEfSe) identified discriminative taxa between uninfected, epithelial hyperplasia, adenomatous hyperplasia, and carcinoma groups (LDA score >3, *p* < 0.05). Functional profiles were predicted from 16S data using PICRUSt2 v2.3.0f and mapped to Clusters of Orthologous Genes using the Wilcoxon rank-sum test. Criteria for core microbiota were the equally weighted average relative abundance over 0.1% and equally weighted average frequency of occurrence over 30%.

### Statistical methods of immunohistochemistry

For the statistical analysis of immunohistochemistry results, we utilized Image-Pro Plus 6.0 software to assess the positive staining within tissue sections quantitatively. The intensity of proliferating cell nuclear antigen (PCNA) staining was quantified using the integrated optical density (IOD) normalized to the area of staining (IOD/area). A Student’s *t*-test was employed to determine the statistical significance of the differences observed between groups. A *p*-value less than 0.05 was considered statistically significant.

## Results

### Sample collection and pathological classification

**Figure 1:**
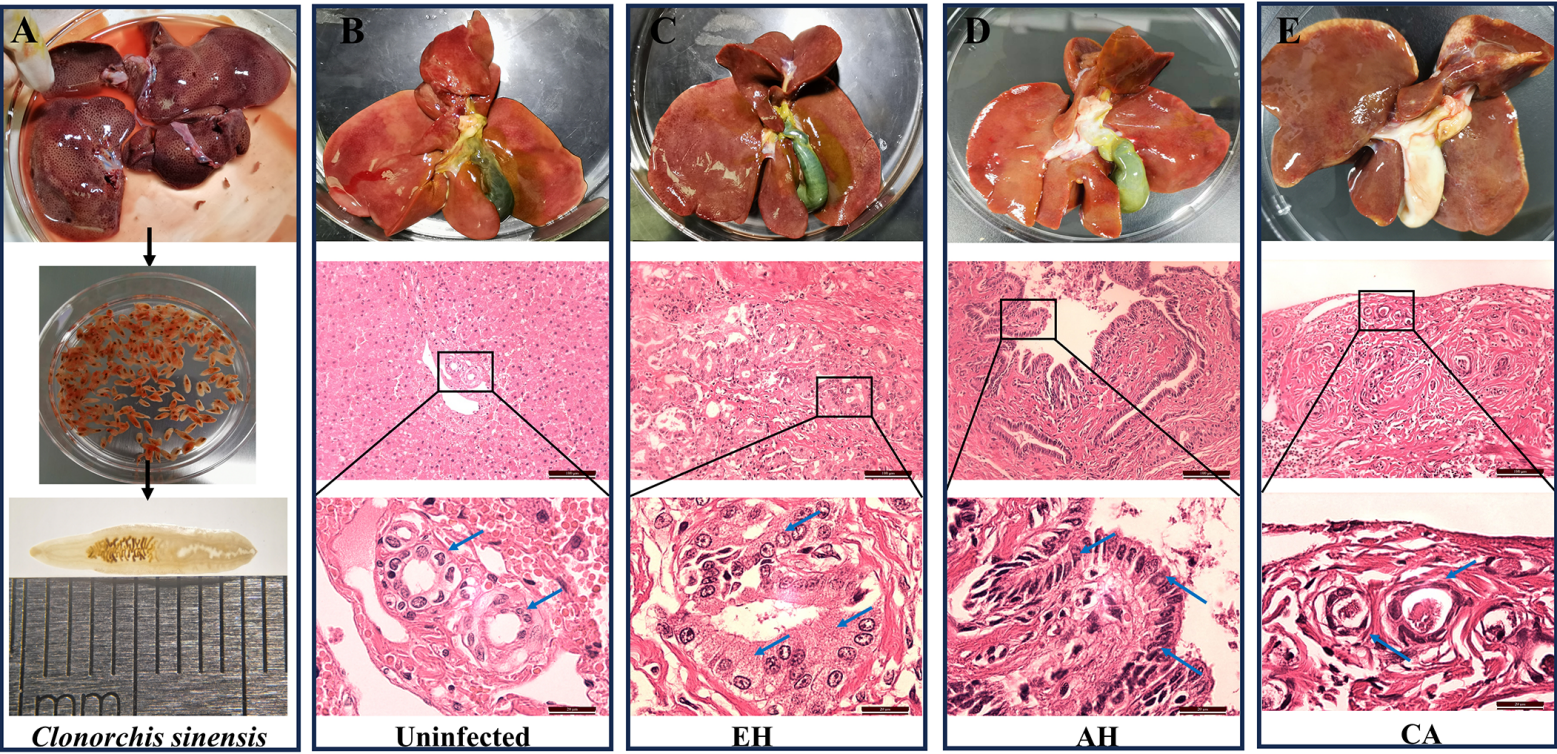
Histological examinations using hematoxylin and eosin (HE) staining to observe the impact of *C. sinensis* infection on feline bile duct epithelium. A: Intravital collection and morphological observation of *C. sinensis.* B: HE staining of intrahepatic bile duct tissue from the uninfected group. C, D, and E present the HE staining analysis of intrahepatic bile duct tissues from cats infected with *C. sinensis*, illustrating epithelial hyperplasia (EH), adenomatous hyperplasia (AH), and carcinoma (CA), respectively. The blue arrows in panels C, D, and E point to the typical bile duct cells under various pathological conditions within the intrahepatic bile duct tissue.

In this study, we conducted a comprehensive observation of 112 feline livers sourced from Nanning City, China. *Clonorchis sinensis* infection was identified in 59% (66/112) of the samples, as shown in Figure 1A. Based on the selection criteria detailed in the methods section, 26 of these cases were further selected for in-depth examination. These cases were subsequently divided into two distinct groups for comparative analysis. Histological analysis using HE staining revealed no abnormalities in the bile ducts of the uninfected group (Fig. 1B). In contrast, the infected group displayed progressive histopathological changes corresponding to increasing severity of clonorchiasis (Fig. 1C–E). Specifically, in the early stages of the disease, the overall liver morphology of the infected and uninfected groups appeared similar; however, there was a significant increase in the number of bile duct epithelial cells in the infected group, with individual cells being slightly larger than normal cells (Fig. 1C). As the disease progressed, adenomatous hyperplasia (AH) occurred. The hepatic duct wall thickened significantly, and there was mild nuclear pseudostratification with an increased nuclear-to-cytoplasmic ratio and nuclear elongation (Fig. 1D). Finally, in late-stage conditions, cholangiocarcinoma (CA) developed in three cats, characterized by discernible gray and solid masses in the liver. The predominant feature was characterized by the tubular growth of varying sizes, accompanied by heightened cellular heterogeneity and a higher nuclear-cytoplasmic ratio (Fig. 1E).

### Immunohistochemistry and histopathology of cat liver

**Figure 2:**
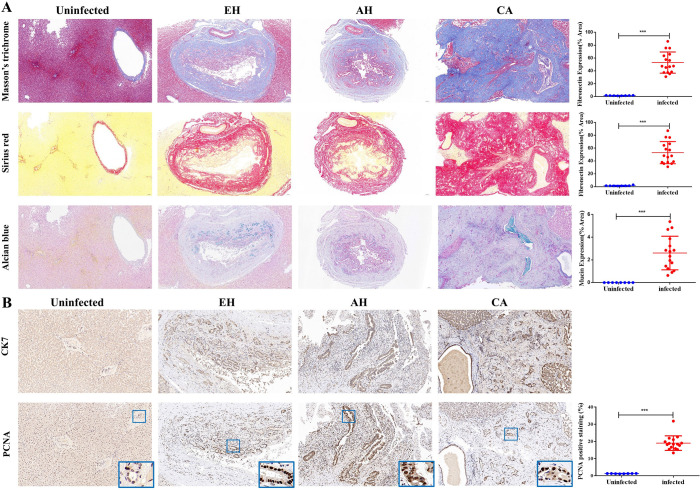
The histopathological examination of hepatobiliary tissues in cats. A: Histopathological analysis using Masson’s trichrome, Sirius red, and Alcian blue staining techniques to analyze the intrahepatic bile duct tissues of cats, including the analysis of the area proportion of positive staining. B: Immunohistochemical staining of intrahepatic bile duct tissues of cats using CK7 and PCNA, analyzing the IOD/Area value for PCNA positive expression. The samples were examined at 200× magnifications. Statistical significance: *p < 0.05; **p < 0.01; ***p < 0.005; ****p < 0.001.

The Masson’s trichrome and Sirius red staining techniques were utilized to analyze the extent of fibrosis and collagen deposition in cat livers (Fig. 2A). In the uninfected group, no apparent pathological changes were observed. However, the infected group exhibited a significant increase in collagen fibers surrounding the bile duct, particularly in the CA group, indicating that *C. sinensis* infection can lead to periductal fibrosis of the bile duct. Alcian blue staining was employed to detect mucin presence in liver slices (Fig. 2A). The infected group displayed faint blue depositions compared to the uninfected group, indicating a significant increase in mucin. Immunohistochemistry results indicated that following *C. sinensis* infection in felines, bile duct cells showed a strong positive response to PCNA antibodies (Fig. 2B). CDK7 specifically marks bile ducts.

### *Clonorchis sinensis* infection and bile microbiota composition and diversity in cat bile

**Figure 3:**
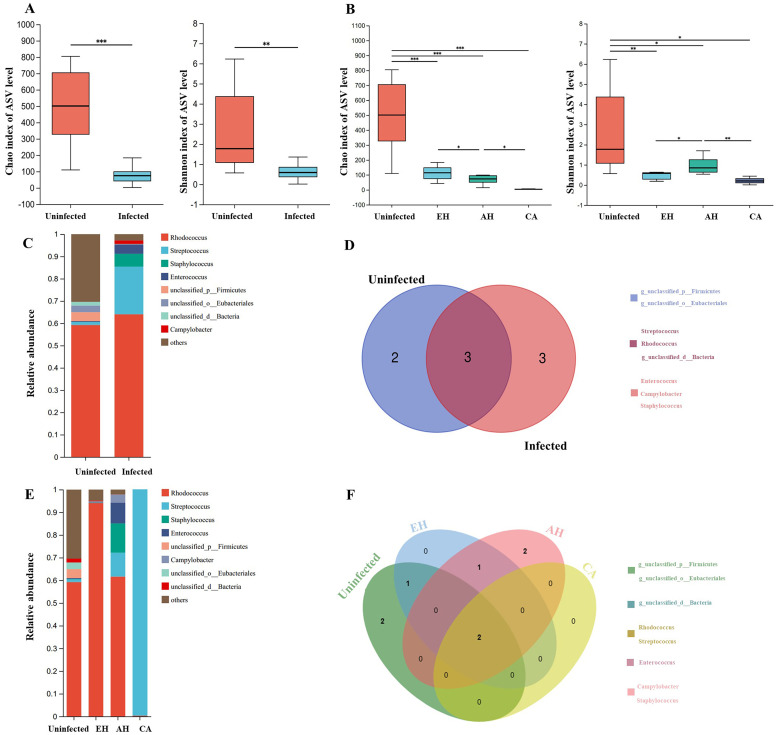
Influence of *C. sinensis* on the diversity and composition of feline bile microbiota. A: Analysis of α-diversity of bile microbiota in uninfected and *C. sinensis*-infected groups. B: α-diversity analysis of bile microbiota across different disease stages of the bile duct for the uninfected and infected groups. C: Composition of bile microbiota at the genus level in uninfected and *C. sinensis*-infected groups. D: Core microbial taxa in uninfected and *C. sinensis*-infected groups. E: Composition of bile microbiota at the genus level in uninfected groups and across different bile duct disease stages for *C. sinensis*-infected group. F: Core microbial taxa in uninfected groups and across different bile duct disease stages for *C. sinensis*-infected group. Statistical significance: *p < 0.05; **p < 0.01; ***p < 0.005; ****p < 0.001.

**Figure S1:**
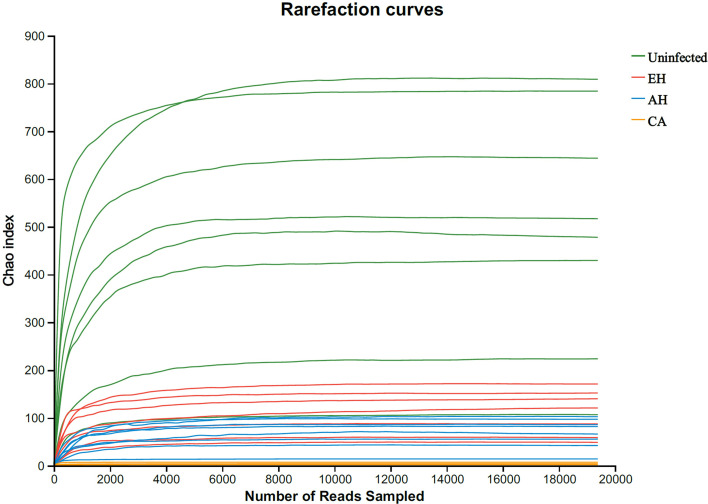
Rarefaction curve analysis conducted on normalized ASVs.

Sequencing generated 753,031 high-quality reads, with 19,384 to 48,018 reads per sample. Taxonomic classification revealed that the sequences represented 27 phyla and 702 genera. Rarefaction curve analysis indicated sufficient sequencing depth to characterize the microbiota (Supplementary Fig. S1). Richness and alpha diversity, assessed via Chao1 and Shannon indices, were significantly lower in the infected versus uninfected cats (Fig. 3A), demonstrating that *C. sinensis* is associated with alterations in the microbial structure. Analysis of alpha diversity across disease stages revealed decreasing richness (Chao1 index) with worsening histopathology. The Shannon index indicated a significant diversity decrease during early epithelial hyperplasia (EH), with a subsequent fluctuating pattern as the disease advanced to adenomatous hyperplasia (AH) and carcinoma (CA) (Fig. 3B).

Composition analysis at the genus level (abundance ≥0.01%) revealed increases in *Streptococcus* and *Staphylococcus* proportional abundance in infected cats, while *Rhodococcus* dominated in both groups (Fig. 3C). Core microbiota analysis identified *Rhodococcus*, *Streptococcus*, and unclassified- Firmicutes and Eubacteriales as core taxa in uninfected cats, while *Campylobacter*, *Enterococcus*, and *Staphylococcus* were unique core members in the infected group (Fig. 3D). In disease stage-specific analysis, *Streptococcus* abundance continually increased, reaching >90% in CA. Unique core taxa were identified in each stage (Fig. 3E, F).

### Progressive microbiota alterations across clonorchiasis disease stages

**Figure 4:**
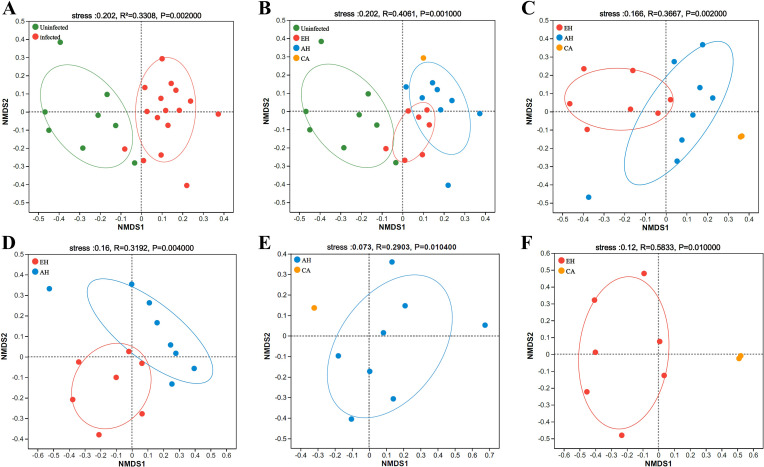
Impact of *C. sinensis* on the β-diversity of the cat biliary microbiota. A: β-Diversity analysis of the biliary microbiota between non-infected and *C. sinensis*-infected groups. B: β-Diversity analysis of the biliary microbiota in the uninfected and different biliary disease stages in *C. sinensis* -infected. C: Biliary microbiota in EH, AH, and CA, three distinct clinical phases of the bile duct caused by *C. sinensis* infection, were analyzed for β-Diversity. D: β-Diversity analysis of the biliary microbiota between the EH and AH groups. E: β-Diversity analysis of the biliary microbiota between the AH and CA groups. F: β-Diversity analysis of the biliary microbiota between the EH and CA groups.

The NMDS analysis revealed a distinct clustering of microbiota profiles from uninfected versus *C. sinensis*-infected cats (Fig. 4A), with statistically significant differences in community structure (PERMANOVA, *p* < 0.05). We next conducted β-diversity analysis to assess microbiota changes across progressive stages of fluke-induced biliary disease. Clear segregation of microbiota composition was observed between the uninfected group and groups representing epithelial hyperplasia (EH), adenomatous hyperplasia (AH), and carcinoma (CA) (Fig. 4B–F). Statistically significant differences in community structure were detected between the uninfected group and the EH, AH, and CA groups (PERMANOVA, *p* < 0.05), as well as between sequential disease stages.

### *Streptococcus* enrichment tracks the progression of clonorchiasis

**Figure 5:**
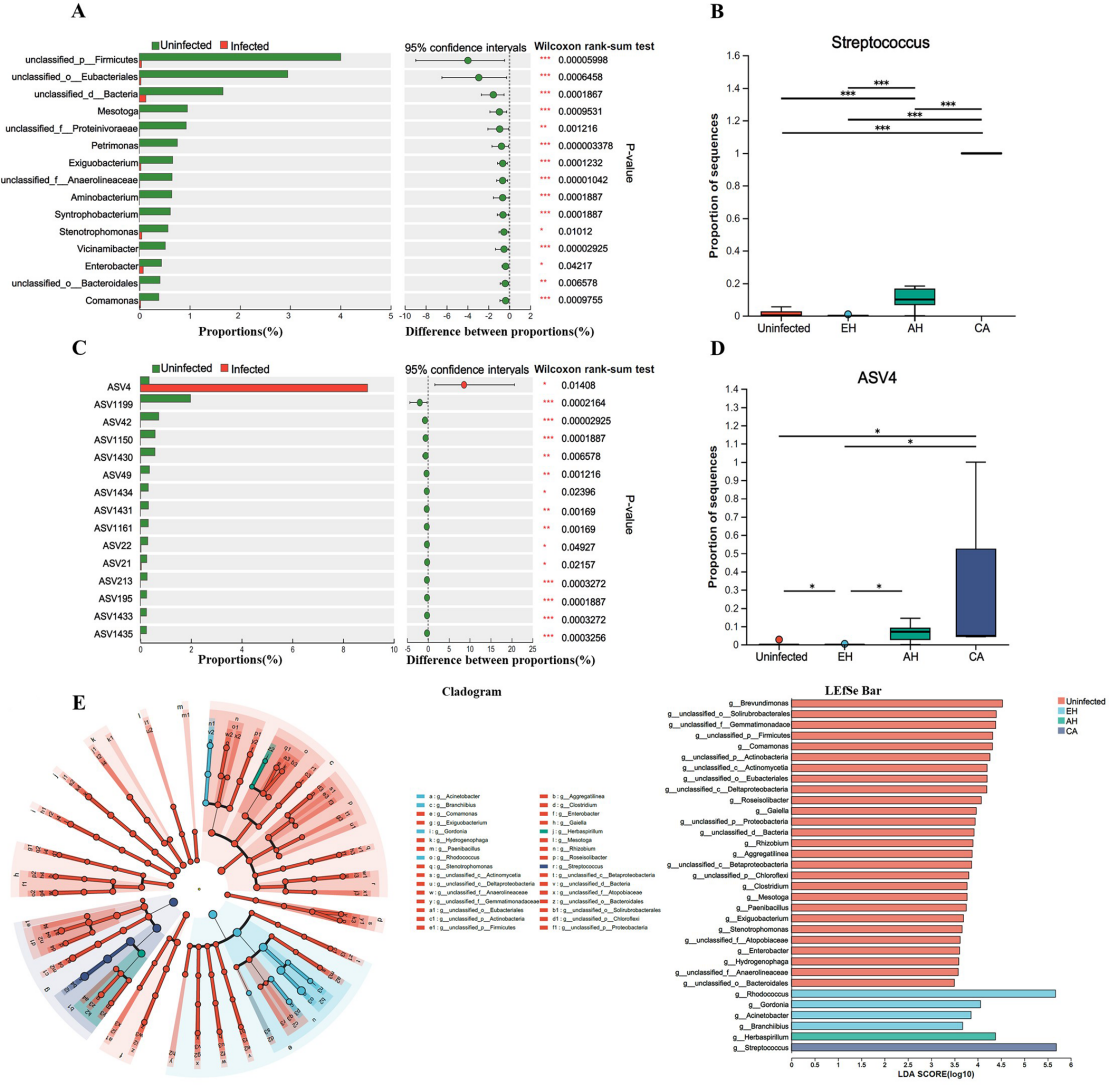
Analysis of microbial species differences in bile. A: Analysis of statistically significant microbial taxa in the bile of uninfected and *C. sinensis*-infected groups at the genus level. B: Kruskal–Wallis test analysis of the relative abundance of Streptococcus in the bile of the uninfected, EH, AH, and CA groups. C: Analysis of statistically significant microbial taxa in the bile of uninfected and *C. sinensis*-infected groups at the ASV level. D: Kruskal–Wallis test analysis of the relative abundance of ASV4 in the bile of the uninfected, EH, AH, and CA groups. E: Identification of dominant bacterial genera in the bile microbiota of the uninfected, EH, AH, and CA groups through LEfSe analysis with a threshold of LDA ≥3.

To identify microbial signatures associated with *C. sinensis*-induced dysbiosis, we first compared taxa abundance between infected and uninfected groups using Wilcoxon rank-sum tests with false discovery rate (FDR) correction. At the genus level, no taxa were significantly enriched in the infected versus the uninfected bile samples (Fig. 5A). However, analysis of amplicon sequence variants (ASVs) revealed an increased abundance of ASV4, classified as *Streptococcus*, in the infected group (Fig. 5C).

Next, we performed linear discriminant analysis effect size (LEfSe) to determine taxa that characterize the microbiota of distinct biliary disease stages. A cladogram illustrated the dominant bacteria within each group (Fig. 5E). In total, 33 differential genera were identified (LDA score >3.0; *p* < 0.05). *Rhodococcus*, *Acinetobacter*, and *Branchiibius* predominated in the epithelial hyperplasia (EH) group, while *Herbaspirillum* was most abundant in the adenomatous hyperplasia (AH) group. *Streptococcus* displayed significantly higher relative abundance in the carcinoma (CA) group compared to other groups. The remaining 28 genera were enriched in the uninfected bile.

The abundance of *Streptococcus* significantly increased from the uninfected group to the AH and CA groups (Fig. 5B). Similarly, the *Streptococcus*-classified ASV4 became enriched in AH and CA versus uninfected bile (Fig. 5D).

### Predicting microbial community functions among the Uninfected, EH, AH, and CA groups

**Figure 6:**
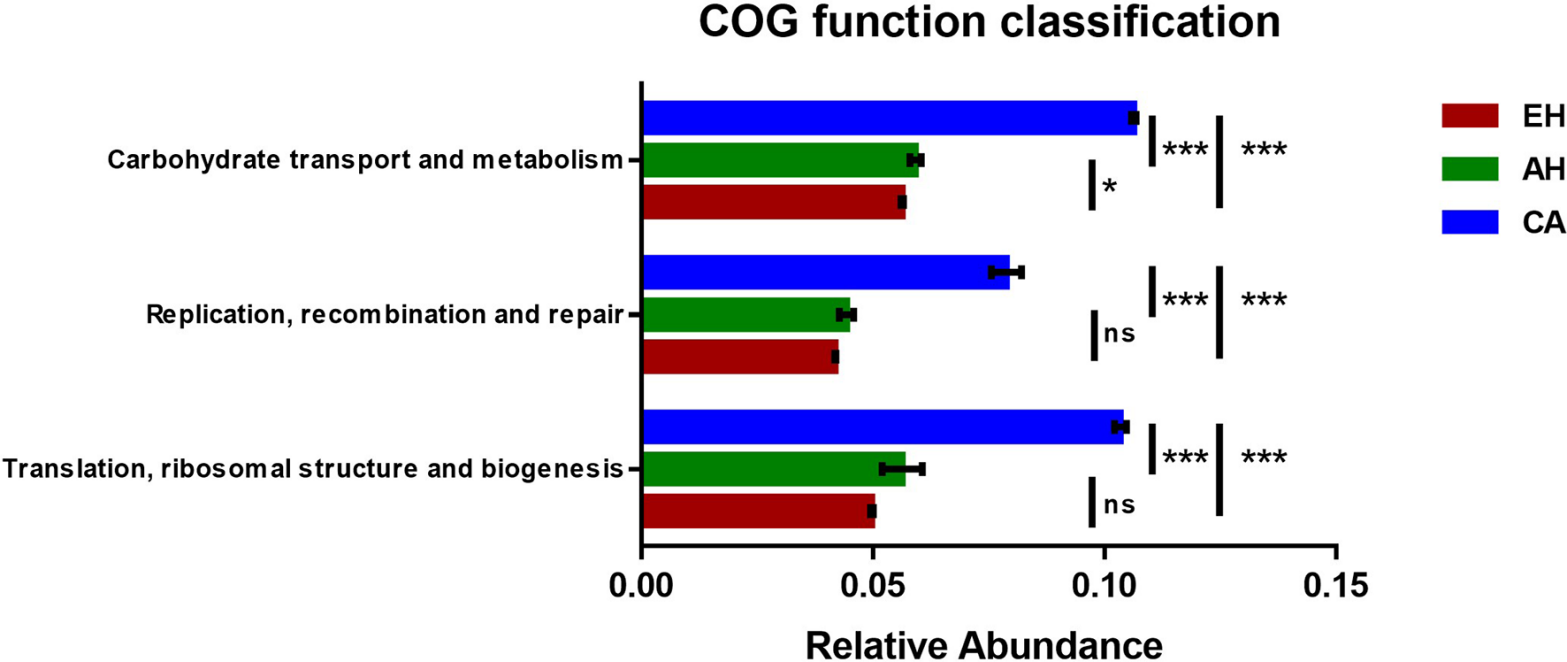
COG functional prediction analysis of gene functionality abundance related to “Translation, ribosomal structure and biogenesis”, “Replication, recombination and repair”, “Nucleotide transport and metabolism”, and “Carbohydrate transport and metabolism” in the EH, AH, and CA groups.

**Figure S2:**
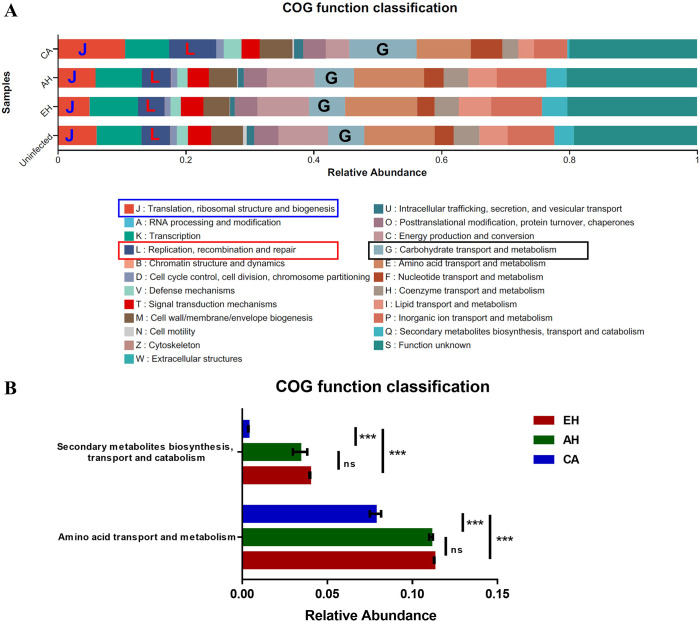
Functional prediction of COG-related gene abundance in the microbiota of the uninfected, EH, AH, and CA groups. (A) Prediction of the functional potential of microbial communities at different stages of biliary disease in the infected and uninfected groups using PICRUSt2. (B) COG functional prediction analysis of gene functionality abundance related to “Amino acid transport and metabolism” and “Secondary metabolite biosynthesis, transport and catabolism” in the EH, AH, and CA groups.

Several cancer-associated pathways were enriched in carcinoma (CA) versus earlier stages, including translation, DNA replication/repair, nucleotide metabolism, and carbohydrate transport and metabolism (Supplementary Fig. S2A). Specifically, carbohydrate transport and metabolism genes progressively increased with advancing biliary disease (Fig. 6). By contrast, amino acid metabolism and secondary metabolite biosynthesis were depleted in CA compared to epithelial hyperplasia (EH) and adenomatous hyperplasia (AH) (Supplementary Fig. S2B).

## Discussion

The pathogenesis of *C. sinensis*-induced cholangiocarcinogenesis is a complex, multi-stage process characterized by initial epithelial hyperplasia, subsequent adenomatous hyperplasia, and eventual carcinoma formation. Transitioning from the effects on epithelial cells, it is crucial to consider the role of the extracellular matrix in this carcinogenic process. Collagen, particularly Type I, is instrumental in maintaining the structural integrity of the tissue matrix. Its significance extends beyond structural support, as Type I collagen has been recognized as a predictive biomarker for intrahepatic cholangiocarcinoma (iCCA) induced by *Opisthorchis viverrini* [37]. Moreover, collagen fibers have been identified as markers for various malignancies, including cholangiocarcinoma [4, 18, 48]. Bile duct obstruction can be accelerated by the release of mucin protein, which in turn can speed up the progression of biliary disorders [52]. In this study, we classified samples into four groups: uninfected, EH, AH, and CA, based on H&E staining. Pathological analysis showed a significant increase in fibrosis, collagen, and mucin protein within the infected groups, particularly in the CA group. Immunohistochemical results indicated significant upregulation in the expression levels of CK7 and PCNA after *C. sinensis* infection in the host. These results demonstrate that *C. sinensis* infection can cause bile duct fibrosis, proliferation, and mucin protein release, which aligns with prior research findings in rodents [38, 54]. Future research should focus on unraveling the molecular pathways associated with these biomarkers and exploring targeted therapeutic interventions for trematode-induced iCCA.

Although previous research has established that *C. sinensis* infection prompts alterations in both the intestinal [19] and biliary microbiota of hosts [6], the specific relationship between these microbiota changes and the progression of bile duct disease in the long term remains poorly understood. In this study, we utilized high-throughput 16S rRNA gene sequencing to characterize alterations in the biliary microbiota during pathological transformation of feline biliary tissues infected with *C. sinensis*, aiming to elucidate the microbiota’s role in this process. Our findings indicate that *C. sinensis* infection leads to decreased richness and diversity of the biliary microbiota, with microbial richness declining further as biliary disease advances. This aligns with previous studies demonstrating reduced biliary microbiota diversity as biliary disease advances, with microbiota diversity lowest in dysplasia or cholangiocarcinoma [24, 34, 35]. However, some inconsistencies exist regarding alpha diversity changes post-infection across different host and fluke models [33, 36, 43], likely reflecting complex microbiota-host-pathogen interactions. β-diversity analysis demonstrated that *C. sinensis* infection is related to marked alterations in bile duct microbiota community structure, which continues to diverge from the normal composition as biliary disease advances from early hyperplastic changes to carcinoma. The stepwise changes in microbial enrichment support a role for dysbiotic microbiota in promoting carcinogenesis during chronic clonorchiasis.

**Figure S3:**
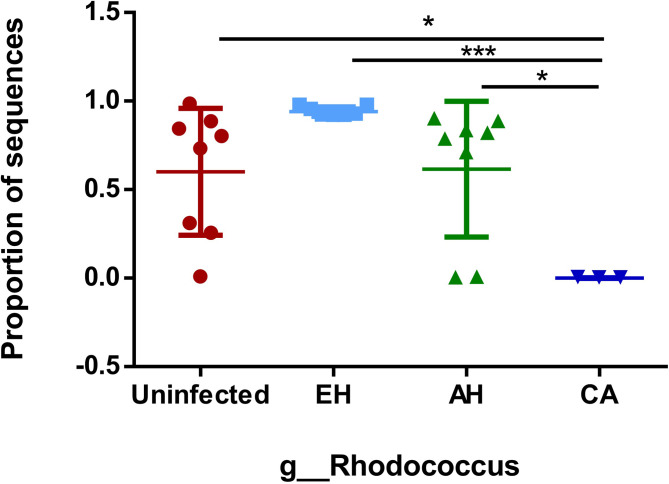
Kruskal–Wallis test analysis of the relative abundance of *Rhodococcus* in the bile of the uninfected, EH, AH, and CA groups.

Notably, we found that the *Streptococcus* genus became significantly enriched in host bile following *C. sinensis* infection, comprising up to 99% of the cancerous bile microbiota. This indicated that *C. sinensis* infection may induce the expansion of specific microbial taxa like *Streptococcus* as biliary lesions advance, highlighting candidate bacteria that may contribute to dysbiosis-promoted carcinogenesis. These results concur with several reports of *Streptococcus* dominance in primary sclerozing cholangitis and biliary tract cancer microbiota [15, 34, 42, 53]. However, studies in hamster models have shown variable impacts of liver fluke infection on *Streptococcus* abundance [17, 19, 33], potentially attributable to differences in host species, infection intensity, and environmental microbes. Our real-world feline samples likely represent a more complex microbiota than laboratory hamsters. The predominant *Streptococcus* species was identified as *S. canis*, an opportunistic zoonotic pathogen rarely causing severe disease in humans [20, 21, 27, 30, 31, 51, 57]. Its presence suggests that *C. sinensis* secretions may promote *S. canis* growth and colonization, and this change in the microbiota favoring *S. canis* may also occur through many other (yet understood) mechanisms. We also observed declining *Rhodococcus* abundance with infection (Supplementary Fig. S3), which may disrupt bile absorption and anti-cancer capabilities given its roles in cholesterol metabolism and biosynthesis of anti-cancer agents [1, 16, 47, 49].

Alterations in microbial community structures can lead to changes in microbial function and metabolism, with metabolic outputs directly influencing tumorigenesis [46]. Prior research has demonstrated that enhanced abundances of microbial functions related to ribosome synthesis [2, 8, 23], carbon metabolism [7, 13], and replication/recombination/repair [22, 25] can promote cancer onset and progression. However, one study found that *Opisthorchis viverrini* infection in cholangiocarcinoma patients significantly increased predicted microbial amino acid metabolism compared to uninfected tissue [9]. This highlights potential differences in mechanisms underlying cholangiocarcinogenesis induced by various parasites. In our study, *C. sinensis* infection of cat hosts increased the abundance of microbial genes related to carbohydrate transport/metabolism as bile duct lesions progressed. Additionally, genes associated with cancer-related functions, including translation, DNA repair, and nucleotide metabolism, became more abundant. These predicted functional shifts indicate that *C. sinensis*-induced changes in bile duct microbiota may promote carcinogenesis through the modulation of microbial metabolic pathways. The stepwise enrichment of carbohydrate transport and metabolism pathways suggests these microbe-mediated processes may be particularly relevant for biliary lesion progression in clonorchiasis. Further studies validating the altered gene content and metabolite profiles are warranted to establish microbe-host interactions facilitating dysbiosis-driven biliary carcinogenesis. These observations suggest that parasites may alter the biliary microbial structure to cause functional shifts that progressively deteriorate bile duct diseases, and this change can also be mediated via other cellular, immunological, or biochemical pathways.

Our study revealed dynamic bile microbiota changes during *C. sinensis-*induced biliary disease progression with specific microbial signatures potentially linked to severity. Some limitations exist. For example, the small sample size from limited geographic regions may incompletely capture microbial community shifts in the cancer group. In addition, a comprehensive approach integrating host genetics (e.g., transcriptomics of cholangiocarcinoma tissues), functional outputs (e.g., bile metabolomics), and environmental factors (e.g., microbiome profiling of *C. sinensis*-infected tissues) is necessary to dissect the contributions of the host and microbiome to tumorigenesis. This highlights the need for more extensive multi-omics studies to fully elucidate dynamic interactions between the parasite, host, and microbiome underlying fluke-associated cholangiocarcinogenesis.

## Conclusion

This work establishes an important foundation for further research into the complex interplay between parasites, biliary microbes, and the liver in fluke-induced carcinogenesis. Identification of microbiota signatures associated with deteriorating biliary health advances our mechanistic understanding of helminth-induced carcinogenesis.

## Supplementary material

The supplementary material for this article can be found at https://www.parasite-journal.org/10.1051/parasite/2024053/olm.
